# Identification of causative fungus from sterile abscess using metagenomics followed by in situ hybridization

**DOI:** 10.1099/acmi.0.000779.v3

**Published:** 2024-08-15

**Authors:** Hiroya Oki, Ryotaro Niwa, Somboonthum Pranee, Daisuke Motooka, Yoshiyuki Onda, Jun Nakata, Hiroko Nakajima, Yoshihiro Oka, Haruo Sugiyama, Yuka Yoshii, Naoyuki Anzai, Shota Nakamura, Tetsuya Iida

**Affiliations:** 1 Department of Infection Metagenomics, Research Institute for Microbial Diseases (RIMD), Osaka University, Osaka, Japan; 2 NGS Core Facility, Research Institute for Microbial Diseases (RIMD), Osaka University, Osaka, Japan; 3 Department of Hematology and Oncology, Takatsuki Red Cross Hospital, Osaka, Japan; 4 Department of Hematology, Yodogawa Christian Hospital, Osaka, Japan; 5 Department of Bacterial Infections, Research Institute for Microbial Diseases (RIMD), Osaka University, Osaka, Japan; 6 Institute for Open and Transdisciplinary Research Initiatives (OTRI), Integrated Frontier Research for Medical Science Division (iFremed), Osaka University, Osaka, Japan; 7 Department of Hematology and Oncology, Osaka Red Cross Hospital, Osaka, Japan; 8 Department of Clinical Laboratory and Biomedical Sciences, Graduate School of Medicine, Osaka University, Osaka, Japan; 9 Department of Cancer Immunology, Graduate School of Medicine, Osaka University, Osaka, Japan; 10 Department of Cancer Stem Cell Biology, Graduate School of Medicine, Osaka University, Osaka, Japan; 11 Department of Immunopathology, Immunology Frontier Research Center (World Premier International Research Center), Osaka University, Osaka, Japan

**Keywords:** AML, metagenome, NGS, *T. asahii*

## Abstract

**Introduction.** Invasive fungal infections require early diagnosis for treatment. Microscopic observation of biopsy and blood culture is the gold standard for the identification of the causative fungus, but it is difficult to identify the causative pathogen by a sterile abscess biopsy.

**Case Presentation.** We present a case report of breakthrough invasive trichosporonosis in a 65-year-old Japanese male with acute myeloid leukaemia receiving antifungal prophylaxis. Blood cultures showed no fungal growth, and a liver biopsy and a removed spleen with abscess showed fragmented fungi, but no fungal identification was possible. This report demonstrates that retrospective analyses were able to identify the causative fungus.

**Conclusion.** We narrowed down the candidate fungi by deep sequencing of the ITS1 region of fungal genome and confirmed that the fungus observed in the specimen was *Trichosporon asahii* by *in situ* hybridization using a DNA probe targeting 26S rRNA.

## Data Summary

The sequencing data were deposited at NCBI (accession number: PRJNA1054341).

## Introduction

Abscess formation is sometimes observed in leukaemia patients with febrile neutropenia, and the identification of the causative micro-organism is essential for treatment and prophylaxis against recurrence in subsequent anti-leukaemia therapy. However, when the infection is active, invasive examinations cannot be performed due to severe neutropenia and thrombocytopenia. In most cases, as the infection becomes sterile during neutrophil recovery, an abscess biopsy fails to identify the causative micro-organism [1].

Most abscess-forming invasive fungal infections (IFIs) are caused by *Candida* or *Aspergillus*. However, in recent years, with the increasing use of immunosuppressive drugs, infections caused by rare opportunistic fungal pathogens, such as invasive trichosporonosis caused by *Trichosporon asahii*, are being reported [2]. The mortality rate of patients with deep trichosporonosis is higher than that of patients with IFIs caused by *Candida* or *Aspergillus*, at approximately 70 % [3]. As effective antifungal agents differ for each of these causative fungi, prompt identification of the fungus is essential for treatment. Direct observation of fungi in biopsies represents the gold standard for fungal identification [2]. However, due to the limited presence of fungi in biopsy tissue and their complex morphology, distinguishing them can be challenging [2,4, 5]. While blood cultures are also frequently used, they are less sensitive, and causative fungi may not grow in cases where antifungal drugs are being administered [5,6]. Advances in molecular biology have introduced techniques such as polymerase chain reaction and sequence-based methods that target specific genes or regions [7]. However, these techniques are limited to the identification of some common pathogenic fungi, and appropriate detection methods for clinical practice have not yet been established for most rare fungi, including *T. asahii* [7].

The advent of next-generation sequencing (NGS) technology has brought a breakthrough in pathogen identification. NGS-based pathogen identification has led to rapid disease diagnosis, drug resistance profile analysis and identification of novel pathogens. In particular, metagenomic analysis allows for the comprehensive analysis of all DNA present in the genome, allowing for the simultaneous identification of multiple micro-organisms without relying on specific target genes or regions [8,9]. However, when culture amplifies the number of pathogens, alternative identification methods are employed, making the process less cost-effective. Moreover, biopsy specimens are often contaminated with non-pathogenic pathogens from the body and environmental contaminants (during the collection process), leading to the lack of standardized criteria for pathogen identification [8,10]. Here, we identified scenarios and methodologies in which NGS-based pathogen identification proves most beneficial in clinical applications.

## Case presentation

**Fig. 1.**
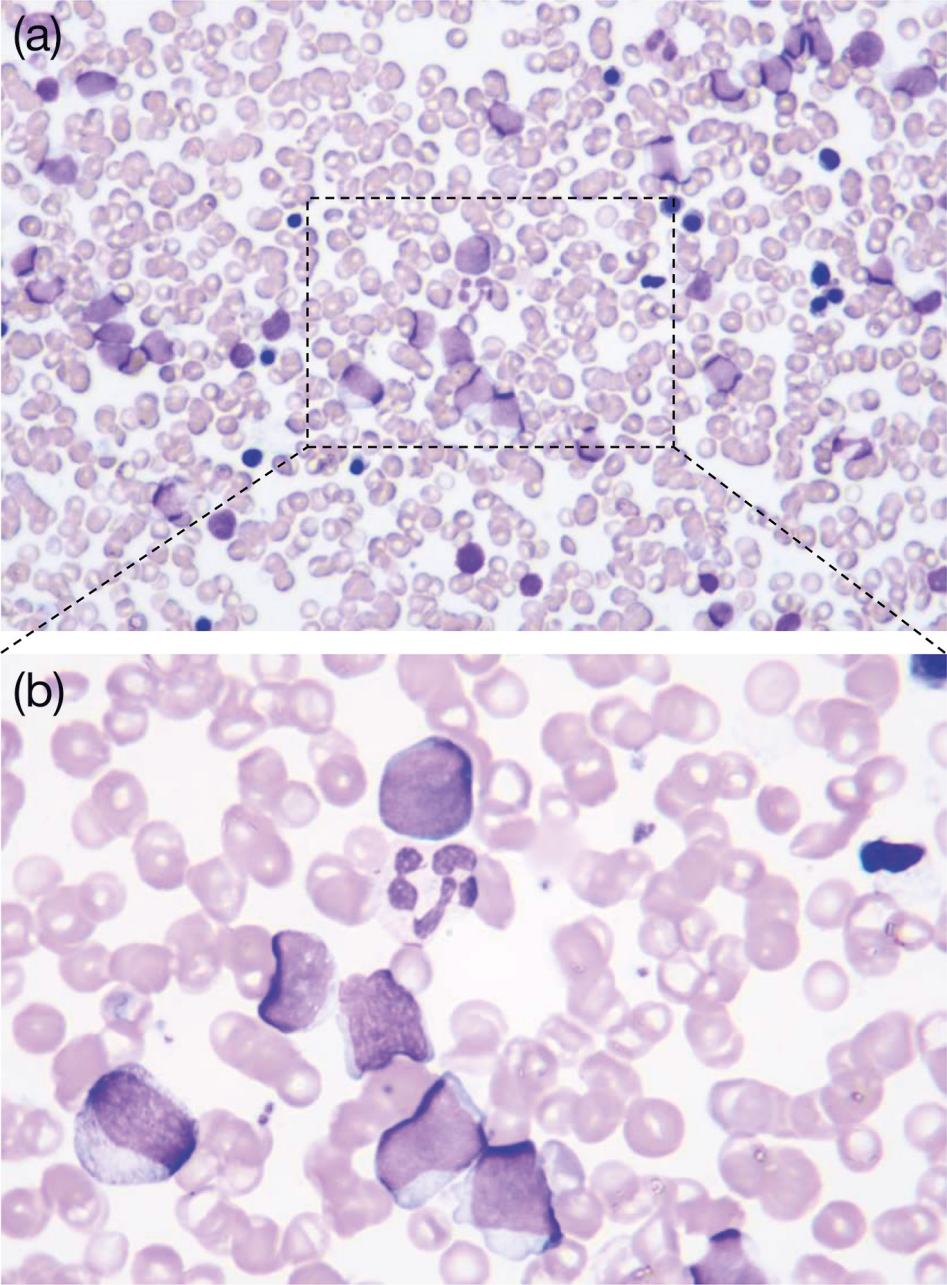
May–Grunwald–Giemsa stains of the patient’s bone marrow aspirate showing myeloblasts with granulocytic differentiation (a: ×80, b: ×20).

**Fig. 2.**
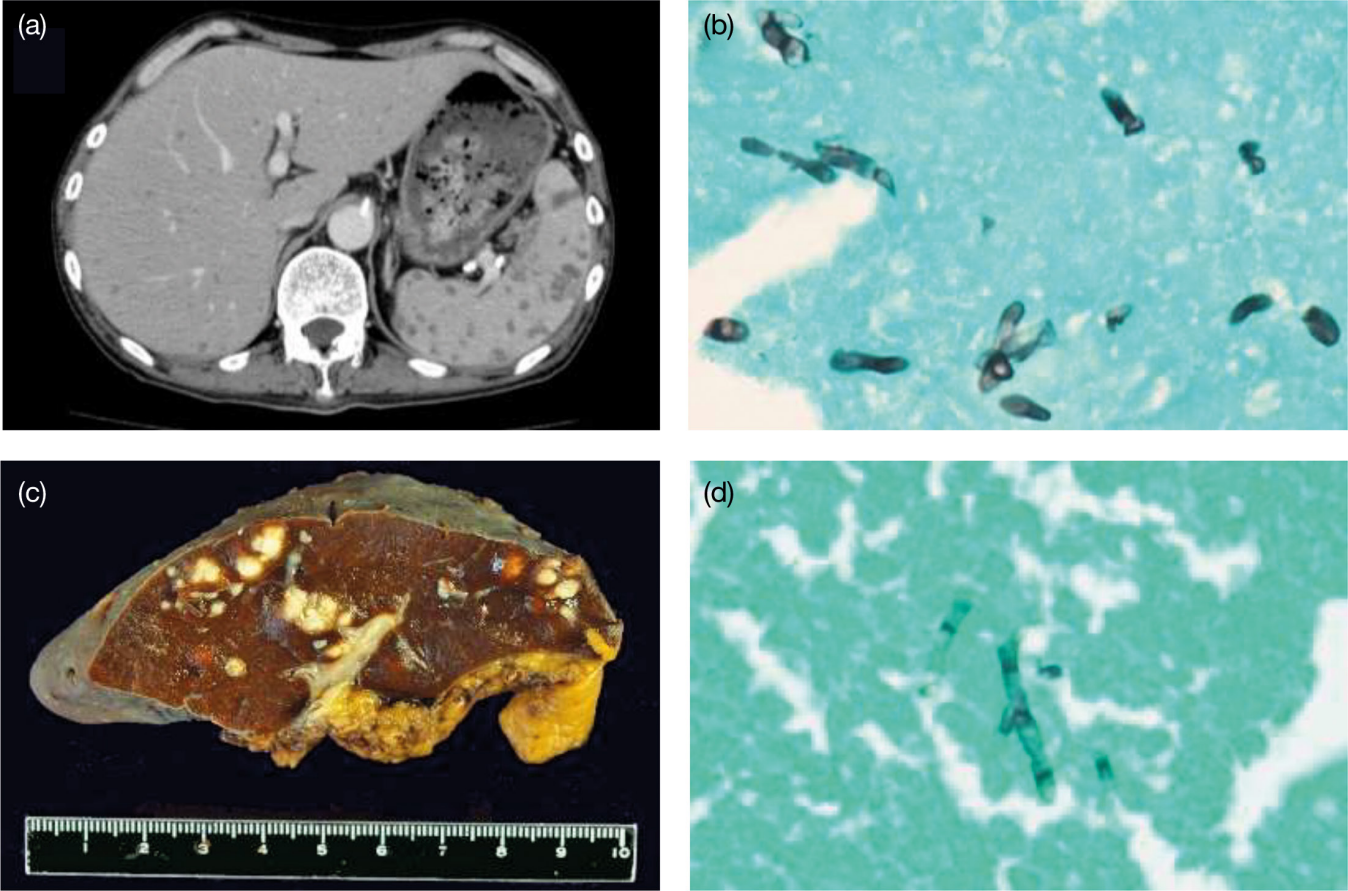
*T. asahii* infection in the patient with AML. (a) Contrast-enhanced computed tomography scan of hepatosplenic abscesses. (b) Liver biopsy stained using Grocott’s methenamine silver (GMS) showing the fungi. (c) Removed spleen showing abscesses. (d) Removed spleen stained using GMS showing fungi similar to those in the liver biopsy.

The patient was a 65-year-old Japanese male with a history of grade 4 chronic kidney failure and benign prostate hyperplasia. He was introduced to our hospital due to neutropenia (white blood cell counts, 1300/µl) and anaemia (red blood cell counts, 334×10^4^/µl). Bone marrow examination revealed 50.5 % myeloblasts with granulocytic differentiation (Fig. 1a, b), and neither leukaemia-causing translocations nor leukaemia-causing gene mutations were detected. Additionally, the mRNA levels of Wilms’ tumour 1 gene, a leukaemia marker, were increased to 420 copies/μg of RNA. Therefore, he was diagnosed with acute myeloid leukaemia (AML) with maturation according to the fourth World Health Organization criteria [11]. The full form of the IA regimen (Idarubicin and cytarabine:AraC) consists of idarubicin 12 mg m^−2^ for 3 days and cytarabine 100 mg m^−2^ for 7 days; however, the dose of idarubicin was reduced to 8 mg m^−2^ for 3 days owing to renal dysfunction. This first induction chemotherapy showed no response. The full form of the MEtA regimen [12] comprises mitoxantrone 8 mg m^−2^ for 2–3 days and etoposide 50 mg/body and cytarabine 30 mg/body for 11–14 days; however, it was modified to mitoxantrone 5 mg m^−2^ for 2–3 days and etoposide 25 mg m^−2^ and cytarabine 15 mg m^−2^ for 10 days owing to renal dysfunction and to avoid severe neutropenia. This second induction therapy reduced myeloblast frequencies and the mRNA levels of Wilms’ tumour 1 gene from 53.3 to 7.8 % and from 230 to 69 copies/μg of RNA, respectively. Therefore, the repeated MEtA regimen was performed 60 days after the second induction chemotherapy, and subsequent hematopoietic stem cell transplantation was planned. However, febrile neutropenia occurred after the chemotherapy. Because the spike fever was not improved by meropenem and vancomycin, fungal infection was suspected. Voriconazole and liposomal amphotericin B were administered instead of oral itraconazole for prophylaxis, but the spike fever continued even after the recovery of neutrophils. Whole-body computed tomography revealed multiple low-density lesions, which were abscesses, in the liver and spleen (Fig. 2a) . To identify the causative micro-organisms, we repeatedly obtained blood cultures, but none revealed any pathogens. No skin lesions were detected throughout the clinical course. Since there have been some cases that show the efficacy of intra-aortic infusion of antifungal agents in patients with hepatosplenic abscesses when neither drainage nor resection of the organs are feasible [13,14], we conducted a splenectomy to reduce the fungal load followed by intra-aortic infusion of micafungin 300 mg/day into the hepatic artery combined with oral itraconazole (200 mg). The patient finally defervesced, and the liver abscesses appeared to improve. Gross findings and Grocott staining of the spleen showed multiple fungal abscesses (Fig. 2c, d), which were similar to the findings from the liver biopsy, although they were never cultivated. Since the liver abscesses disappeared and mRNA levels of Wilms’ tumour 1 gene increased from below the detection level to 1600 copies/μg of RNA, suggesting the molecular relapse of AML, allogenic hematopoietic transplantation was performed with the prophylaxis of continued intravenous micafungin infusion. However, 6 days after the transplantation, a high fever occurred, and the patient subsequently died of multiorgan failure. The blood cultures confirmed the presence of fungemia of *T. asahii*, whose antifungal sensitivity profile showed resistance to micafungin (Table 1).

**Table 1.** Sensitivity profile of isolated *T. asahii* to antifungal agents

Antibiotics	MIC *
Amphotericin B	2.0
5-Fluorocytosine	2.0
Fluconazole	1.0
Itraconazole	0.12
Miconazole	0.12
Micafungin	>16
Voriconazole	0.03
Caspofungin	8

*MIC: minimum inhibitory concentration.

**Fig. 3.**
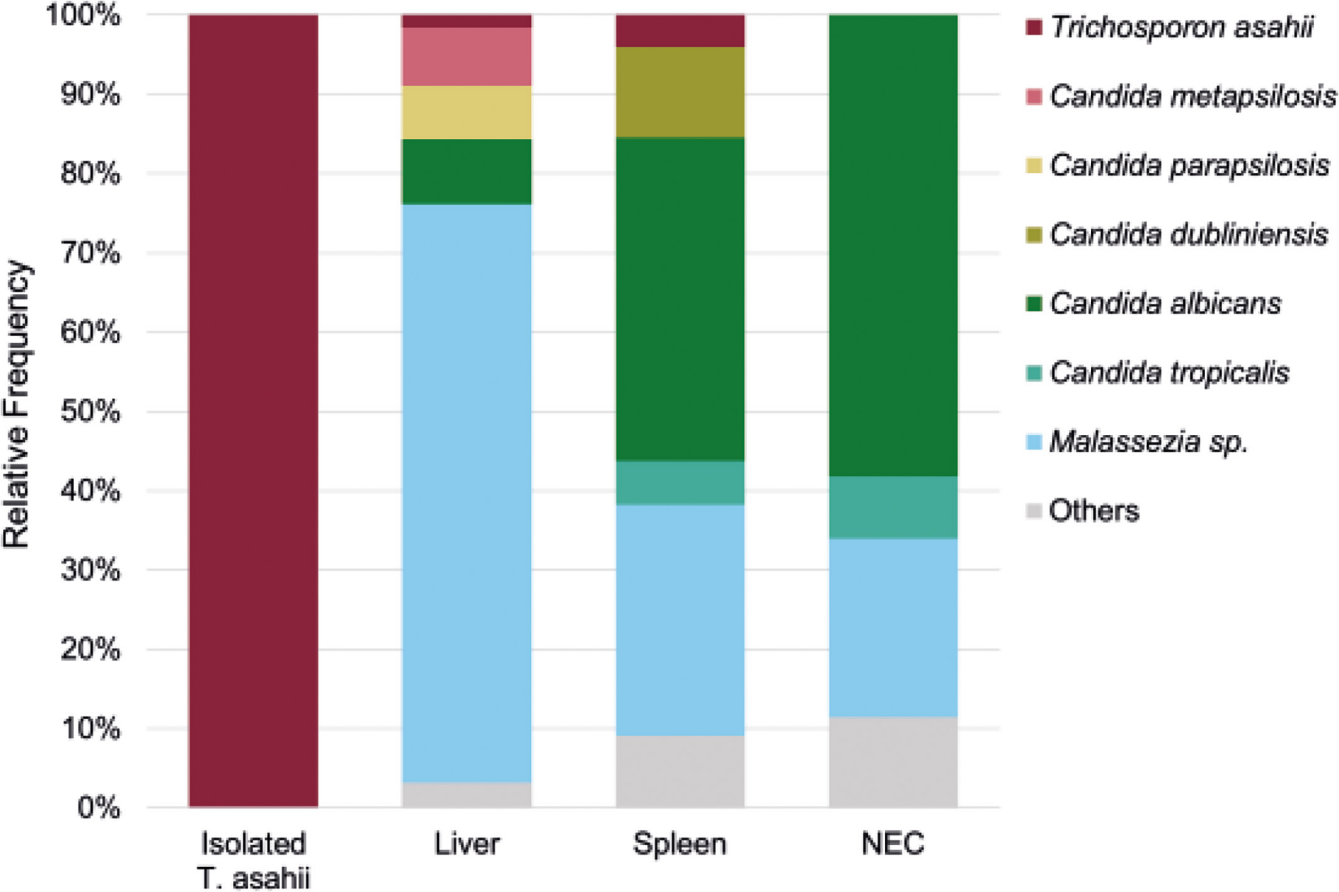
Relative abundance of the fungal taxon in the *T. asahii* isolated from blood culture, liver biopsy, removed spleen and an NEC as a working control.

**Fig. 4.**
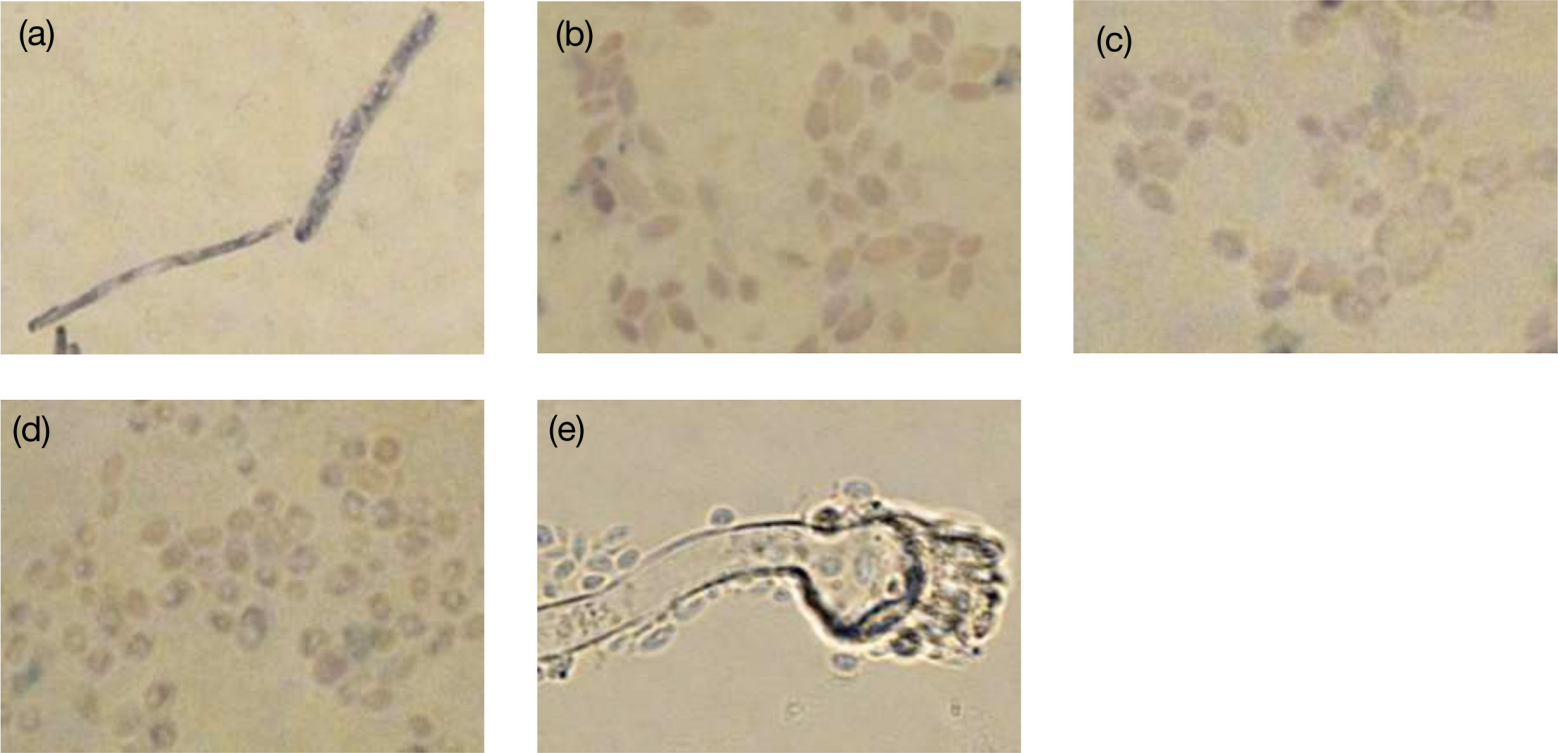
*In situ* hybridization of cultured fungi using a biotinylated DNA probe targeting the 26S rRNA sequence of *T. asahii* (5′-GTCTCCTGGAAAGGAGTAT-3′). (**a**) *Trichosporon asahii* (NBRC 103889). (**b**) *Candida albicans* (NBRC 1385). (**c**) *Candida dubliniensis* (NCPF 3949). (**d**) *Candida parapsilosis* (NBRC 0708). (e) *Aspergillus terreus* (NBRC 33026).

**Fig. 5.**
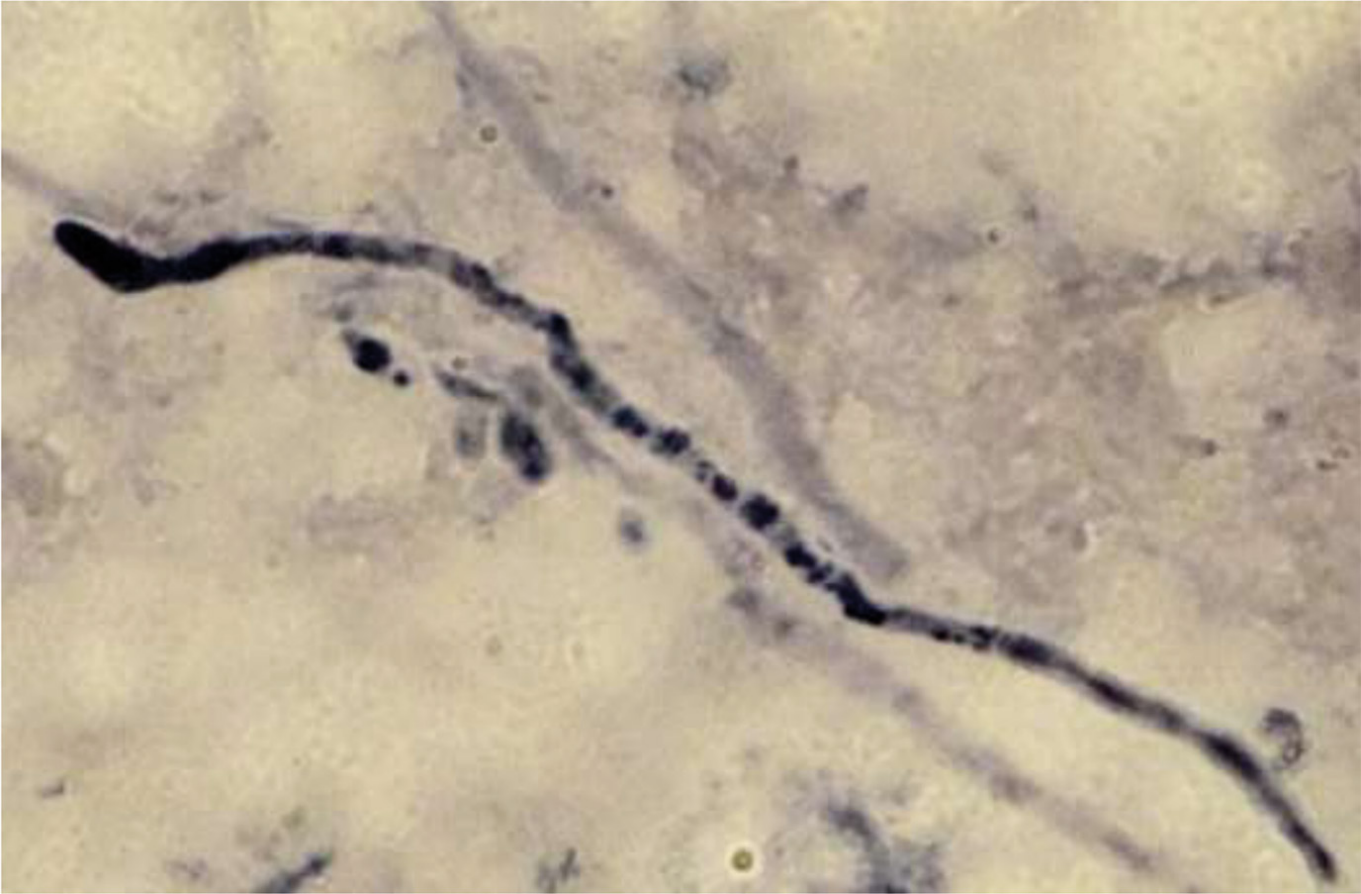
*In situ* hybridization of fungi in an abscess from the spleen using a biotinylated DNA probe targeting the 26S rRNA sequence of *T. asahii* (5′-GTCTCCTGGAAAGGAGTAT-3’).

Even with repeated blood cultures, we were unable to grow the fungus. This could be attributed to the low numbers of fungi, which exhibited reduced biological activity, observed in the liver biopsy and spleen. Additionally, continuous administration of voriconazole and liposomal amphotericin B to the patient even during blood culture examination may have contributed to this outcome. In this study, we conducted a retrospective metagenomic analysis to determine whether the causative fungus *T. asahii* could be identified from biopsy specimens that were unsuitable for pathogen identification. DNA was extracted from fungi isolated from various sources, including blood cultures, residual liver biopsies (in storage) and excised spleens. We used distilled water as the negative extraction control (NEC). All of these NGS experiments were performed at the NGS Core Facility at Osaka University. Deep sequencing of the fungal ITS1 region was performed using each extracted sample. *T. asahii* was detected in all samples except the NEC (Fig. 3). However, more than 80 % of the reads obtained from liver biopsies and spleen specimens corresponded to *Candida* spp. and *Malassezia* sp. The inclusion of the NEC to the samples helped narrow down potential pathogenic fungi to three in the liver biopsies and two in the spleen. Of these, *Candida* spp. were detected only in the liver biopsy or spleen. In the present case, blood samples were always negative for *Candida* antigen; therefore, *Candida* spp. were excluded as candidate causative fungi. Notably, *T. asahii* was the only fungus detected in both the spleen and the liver biopsies but undetected in NEC. To confirm the presence of *T. asahii* in the observed fungi from the liver and spleen biopsies, we designed a biotinylated DNA probe to target the 26S rRNA sequence of *T. asahii* (5′-GTCTCCTGGAAAGGAGTAT-3′). The probe did not stain *Candida* spp. detected in the NGS experiments or *Aspergillus* sp., a common causative agent of IFIs, but specifically stained cultured *T. asahii* (Fig. 4). *In situ* hybridization experiments using the highly specific probe indicated that the fungus observed in the spleen was *T. asahii*. This definitive outcome confirmed the diagnosis of abscesses caused by *T. asahii* (Fig. 5).

## Discussion

Metagenomic analysis has great potential for diagnosing infectious diseases because it can detect all potential pathogens with high sensitivity [8]. However, clinical specimens are often contaminated with endemic skin bacteria and fungi during sample collection, which often make up the majority of the data obtained, as was the case in this study [10]. In addition, contaminants ‘kitome’ in DNA extraction kits and DNA library preparation reagents can mask the DNA information of causative pathogens, which are present only in small quantities. To improve the identification accuracy of the causative pathogen, it is important to use negative controls for library preparation [15]. In this study, we were able to narrow down two and three candidates from the liver and spleen samples, respectively. In the present study, negative controls were prepared in the laboratory; however, samples obtained at earlier time points, such as those obtained in the operating room, could be used to further narrow down the candidates.

In this case, the infection of *T. asahii* was below detection levels by blood cultures and radiology before the transplantation; however, it appeared to be dormant and then resurfaced during neutropenia caused by the transplantation. Therefore, the identification of the causative fungi is essential for determining whether to proceed with the transplantation. IFIs such as those in the present case require early definitive diagnosis and treatment. However, it is often challenging to identify pathogenic fungi from blood cultures or biopsy observations. In addition, the recent heavy use of immunosuppressive agents in the treatment of various diseases has led to an increase in fungal-caused infections. Such patients are susceptible to fungal infections that are rarely seen or reported as human pathogens and may be difficult for even experienced mycologists to identify [2]. Combining metagenome sequencing with *in situ* hybridization using probes may allow rapid and accurate identification of the causative fungus, even if the fungus has a small number of cells, is fragmented and dead or has no previous cases. This will facilitate the selection of an effective antifungal agent for the causative fungus and the determination of whether or not intensive treatment with antifungal prophylaxis can be continued.

## Abbreviations

AML: acute myeloid leukaemia
GMS: Grocott’s methenamine silver
IFIs: invasive fungal infections
ITS: internal transcribed spacer
NEC: negative extraction control
NGS: next-generation sequencing
